# Protrusion of a ceramic femoral head through the acetabular metallic cup in total-hip arthroplasty: A case report

**DOI:** 10.1097/MD.0000000000020469

**Published:** 2020-06-12

**Authors:** Ding Zhao, De-Bao Zhang, Dong-Feng Han, Gui-Shan Gu

**Affiliations:** a Department of Orthopedic Surgery; b Department of Emergency Medicine, The First Hospital of Jilin University, Changchun, Jilin, China.

**Keywords:** dissociation, metallosis, total-hip arthroplasty

## Abstract

**Rationale:**

Dislocation, wear, metallosis, and implant loosening are well-known complications of a failed total-hip arthroplasty (THA), and acetabular liner dissociation is an uncommon but catastrophic complication. To our knowledge, this is the first description of metallosis due to acetabular liner dissociation, but not presenting as a result of wear of a metal-on-metal articulation and a polyethylene liner of other articulation.

**Patient concerns:**

We described a 61-year-old man who had a 2-year history of pain in the right groin region after THA. Postoperative period of primary THA was uneventful. However, he did not undergo postoperative follow-up, and often participated in strenuous sports activities including mountain climbing and long-distance running.

**Diagnosis:**

Radiographs demonstrated superior subluxation of the femoral head and direct articulation and abrasion wear of the ceramic femoral head on the cup. Preoperative laboratory data revealed no signs of infection.

**Interventions:**

We performed revision THA using a direct lateral approach with ceramic-on-ceramic hip prosthesis.

**Outcomes:**

Postoperatively, the patient wore a hip orthosis for 6 weeks to prevent dislocation but was allowed full weight bearing. At 1-year follow-up, there was no recurrence of hip pain.

**Lessons:**

Wear of THA components can result in catastrophic failure of the implants and significant soft-tissue metallosis. Therefore, regular postoperative follow-up is necessary for early intervention, even in those with asymptomatic hips.

## 1 Introduction

Dislocation, wear, metallosis and implant loosening are well-known complications of a failed total-hip arthroplasty (THA).^[1,2]^ Acetabular liner dissociation is an uncommon but catastrophic complication, although some studies have reported this complication with DePuy and Zimmer hip components, due to breakage of the locking system between the cup and liner.^[3]^ After dissociation, wear debris is produced from the bearing surfaces of the hip prosthesis after an extended period.^[4]^ Metallic debris and/or particles from other arthroplasty components, are released by implant wear resulting in metallosis, which is typically recognized upon surgical revision of a prior arthroplasty by the accumulation of black periarticular soft tissues.^[5]^

Here, we report a case of a failed ceramic head and polyethylene liner in a patient with metallosis, and discuss the probable mechanism. To our knowledge, this is the first description of metallosis due to acetabular liner dissociation, but not presenting as a result of wear of a metal-on-metal articulation and a polyethylene liner of other articulation.

## 2 Case report

**Figure 1:**
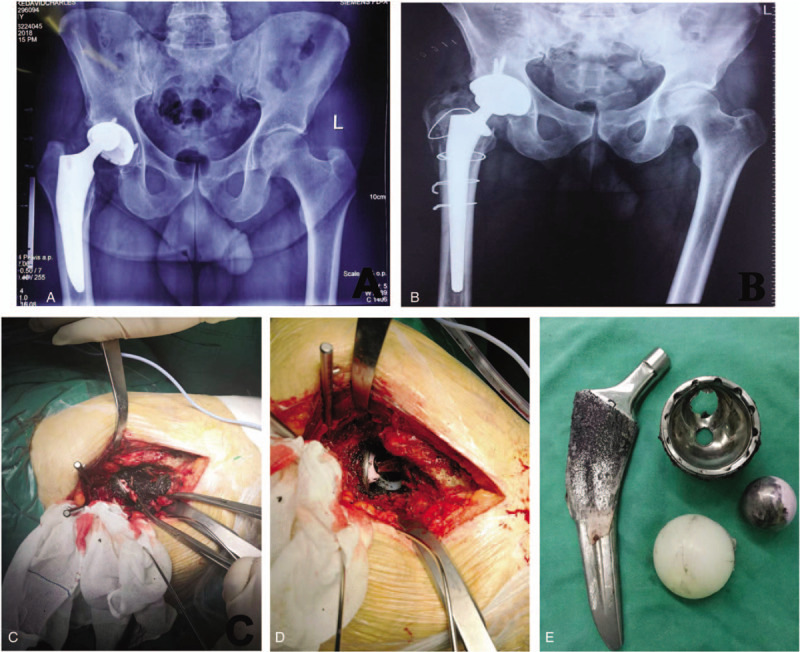
(A) Preoperative anteroposterior pelvis radiographs of the pelvis. (B) Postoperative anteroposterior pelvis radiograph following revision. (C) Copious amount of periprosthetic tissues presenting extensive metallosis. (D) The dissociated acetabular liner in surgery. (E) The destructed metallic shell and the protruded ceramic head through the defect.

A 61-year-old American man complained of right hip pain during his visit on August 24, 2018. He had undergone a right-sided THA in St Peter's clinic in 2010 because of osteoarthritis (OA) of the right hip. In THA, a nonmodular neck implant and femoral stem with proximal titanium porous coating were used (50-mm acetabular cup with a polyethylene liner: Apex BS; size 8 femoral stem: Trilock BPS; 28-mm femoral ceramic head: BIOLOXdelta; DePuy Orthopaedics, Warsaw, IN). Postoperative period was uneventful. However, he did not undergo postoperative follow-up, and often participated in strenuous sports activities including mountain climbing and long-distance running. Since early 2017, he had been complaining about mild pain in the right groin region. By June 2018 the pain had worsened in duration and intensity, limiting his activities of daily living. Radiographs revealed internal superior subluxation of the head within the cup (Fig. 1A). Preoperative laboratory data revealed no signs of infection.

During surgery, performed using a direct lateral approach, we found black pigmentation of metallic debris within the osteolytic lesions and around the joint capsule and acetabulum (Fig. 1C). At the same time, the polyethylene liner was pulled down, and the lower portion that was impinged with the femoral neck was worn out; most of the antirotation locking tabs at the periphery of the polyethylene rim were fracture (Fig. 1D). Furthermore, the ceramic femoral head was in direct contact with the metal acetabular cup, the top of the cup had worn out, and the ceramic femoral head was in good shape (Fig. 1E).

The osteolytic lesions, synovial tissue, and joint capsule were debrided to remove the metal debris to the maximum possible extent, and all parts of the hip prosthesis were replaced. However, for stem removal, a proximal femoral diaphyseal osteotomy was performed.

A 54-mm acetabular cup with a ceramic liner (Pinnacle), a size 11 femoral stem (Corail), and a 36-mm femoral ceramic head (Biolox) (DePuy Orthopaedics) were placed after removing the old components (Fig. 1B). Postoperatively, the patient wore a hip orthosis for 6 weeks to prevent dislocation but was allowed full weight bearing. At 1-year follow-up, there was no recurrence of hip pain.

### 2.1 Institutional review board statement and patient consent statement

The study complied with the Declaration of Helsinki and was approved by the Institutional Review Board of the First Hospital of Jilin University. Patient has provided informed consent for publication of the case.

## 3 Discussion

The main factor limiting long-term THA survival is wear debris production from the bearing surfaces of hip prosthesis after an extended period, including polyethylene, ceramics, and metal.^[6,7]^ Metallosis, is a rare complication after bearing surface wear, which is characterized by extensive amount of metallic debris and the subsequent release of the metallic ions in the soft-tissues surrounding the hip joint. The primary symptoms of patients are discomfort around the groin and gait alterations. In our patient, the symptoms of discomfort around the groin started at 7 years after first THA. He did not present for follow-up after the first surgery, thus preventing the doctors from making an early diagnosis of the wear. Additionally, the extreme load burden on the joint due to sports could have contributed to the severe wear.

All modular acetabular designs have the possibility of liner dissociation with locking mechanism failure.^[8]^ Liner dissociations occurring in THA are likely to be the result of inadequate seating of the polyethylene liner, acetabular designs, liner-cup mismatch, the fragile locking tines, femoral neck impingement against the polyethylene liner.^[3,9]^ To avoid this condition, orthopedic surgeons need to be vigilant during THA to ensure that the angles of acetabular cup are within the Lewinnek safe zone, the locking mechanism is fully engaged without soft-tissue entrapment, and femoral neck impingement is avoid.

Yun et al^[3]^ described 23 cases of DePuy acetabular liner dissociations with no history of trauma, and radiographs demonstrated superior subluxation of the femoral head. In addition, they found fractures of the 3 antirotation tabs at the polyethylene rim, allowing for rotational liner displacement and subsequent direct articulation of the femoral head with the acetabular shell. Consistent with this report, in our patient, superior subluxation of the femoral head was found on radiographs, and most of the antirotation locking tabs at the periphery of the polyethylene rim were fractures.

After dissociation, the polyethylene liner is located at the bottom of the acetabular cup and impinged with the femoral neck, leaving the femoral head prosthesis in direct contact with the inner metal shell of the acetabular cup. Smaller-diameter ceramic femoral heads and larger-diameter acetabular cups create “point-to-face” eccentric wear. In revision surgery, the pinnacle cup was used because of its 10 degree taper-locking mechanism near the equator of the shell and liner derotation tabs for rotational stability.^[10]^

In some reports, BIOLOXdelta ceramic showed a higher rigidity than the pure metal CoCrMo (Lc1: 19.1 vs 2.8 N; Max residual penetration depth: 6.2 vs 12.4 μm).^[11]^ Williams reported that in ceramic-on-metal (COM) hip replacements, where the head is a BIOLOXdelta ceramic and the liner is CoCrMo, wear of COM bearings was primarily metal wear under standard simulator conditions due to the superior hardness of ceramic.^[12]^ Therefore, in our patient, the metal cup had practically been penetrated by the ceramic head, and the ceramic head did not have significant wear. Furthermore, due to the superior hardness of the ceramic, ceramic-on-ceramic bearing couples should be considered to prevent excessive wear in revision THA, including metal debris embedment, as observed in the present case.

In conclusion, wear of THA components can result in catastrophic failure of the implants and significant soft-tissue metallosis. Therefore, regular postoperative follow-up is necessary for early intervention, even in those with asymptomatic hips.

## Author contributions

DZ, DBZ and GSG carried out the surgery. DZ, DBZ and DFH drafted the manuscript. All authors read and approved the final manuscript.
